# Significant differences in written assessments as a result of a blended learning approach used in a clinical examination course in internal medicine: a randomized controlled pilot study

**DOI:** 10.3205/zma001438

**Published:** 2021-02-15

**Authors:** Carolin Sonne, Hasema Persch, Stefanie Rosner, Ilka Ott, Ede Nagy, Christoph Nikendei

**Affiliations:** 1 Technische Universität München, Deutsches Herzzentrum München, Klinik für Herz- und Kreislauferkrankungen des Erwachsenen, Munich, Germany; 2 Universitätsklinikum Ulm, Innere Medizin II, Sektion Sport- und Rehabilitationsmedizin, Ulm, Germany; 3 Universitätsklinikum Heidelberg, Klinik für Allgemeine Innere Medizin und Psychosomatik, Heidelberg, Germany

**Keywords:** blended learning, course on physical examination techniques, app

## Abstract

**Background: **Taking a medical history and performing a physical examination represent basic medical skills. However, numerous national and international studies show that medical students and physicians-to-be demonstrate substantial deficiencies in the proper examination of individual organ systems.

**Aim: **The objective of this study was to conduct a randomized controlled pilot study to see if, in the context of a bedside clinical examination course in internal medicine, an additional app-based blended-learning strategy resulted in (a) higher satisfaction, better self-assessments by students when rating their history-taking skills (b1) and their ability to perform physical examinations (b2), as well as (c) higher multiple-choice test scores at the end of the course, when compared to a traditional teaching strategy.

**Methods: **Within the scope of a bedside course teaching the techniques of clinical examination, 26 students out of a total of 335 students enrolled in the 2012 summer semester and 2012/2013 winter semester were randomly assigned to two groups of the same size. Thirteen students were in an intervention group (IG) with pre- and post-material for studying via an app-based blended-learning tool, and another 13 students were in a control group (CG) with the usual pre- and post-material (handouts). The IG was given an app specifically created for the history-taking and physical exam course, an application program for smartphones enabling them to view course material directly on the smartphone. The CG received the same information in the form of paper-based notes. Prior to course begin, all of the students filled out a questionnaire on sociodemographic data and took a multiple-choice pretest with questions on anamnesis and physical examination. After completing the course, the students again took a multiple-choice test with questions on anamnesis and physical examination.

**Results: **When compared to the CG, the IG showed significantly more improvement on the multiple-choice tests after taking the clinical examination course (p=0.022). This improvement on the MC tests in the IG significantly correlated with the amount of time spent using the app (Spearman’s rho=0.741, p=0.004).

**Conclusion: **When compared to conventional teaching, an app-based blended-learning approach leads to improvement in test scores, possibly as a result of more intensive preparation for and review of the clinical examination course material.

## Zusammenfassung

**Hintergrund: **Die Erhebung der Anamnese und die Durchführung körperlicher Untersuchungstechniken stellen eine grundlegende Kompetenz des ärztlichen Handelns dar. Allerdings zeigen zahlreiche nationale und internationale Studien, dass bei Medizinstudierenden und angehenden Ärzten erhebliche Defizite in der korrekten Durchführung der körperlichen Untersuchung einzelner Organsysteme vorliegen.

**Zielsetzung:** Ziel der vorliegenden Studie war es, in einer randomisiert-kontrollierten Pilotstudie zu überprüfen, ob im Rahmen eines internistischen Bedside-Teaching ein zusätzliches App-basiertes Blended-Learning-Konzept im Vergleich zum klassischen Lehrkonzept (a) zu einer höheren Zufriedenheit, zu einer besseren Selbsteinschätzung der Studierenden bezüglich der Anamneseerhebung (b1) und der Durchführung der körperlichen Untersuchung (b2), als auch (c) zu besseren Multiple-Choice (MC)-Prüfungsergebnissen am Ende des Kurses führt.

**Methodik: **Im Rahmen des internistischen Untersuchungskurses / Bedside-Teaching wurden von insgesamt 335 Studierenden des Sommersemesters 2012 und des Wintersemesters 2012/2013 26 Studierende randomisiert zwei gleich großen Gruppen zugewiesen: 13 Studierende einer Interventionsgruppe (IG) mit Vor- und Nachbereitungsmaterial anhand eines App-basierten Blended-Learning-Konzepts und 13 Studierende einer Kontrollgruppe (KG) mit konventionellem Vor- und Nachbereitungsmaterial (Handout). Die IG erhielt eine auf den Anamnese- und Untersuchungskurs zugeschnitte App, ein Anwendungsprogramm für Smartphones, das es ermöglicht, Lehrmaterial für den Untersuchungskurs direkt auf dem Smartphone einzusehen. Die KG erhielt dieselben Informationen anhand von Skripten. Vor Beginn des Untersuchungskurses füllten alle Studierenden Fragebögen zu soziodemographischen Daten aus und nahmen an einer Eingangs-MC-Klausur mit MC-Fragen zur Anamnese und körperlicher Untersuchung teil. Nach Abschluss des Untersuchungskurses nahmen die Studierenden erneut an einer MC-Klausur mit MC-Fragen zu Anamnese und körperlicher Untersuchung teil.

**Ergebnisse: **Die IG zeigte im Vergleich zur KG eine signifikant höhere Verbesserung in den MC-Klausuren nach Durchlaufen des Untersuchungskurses (p=0,022). Die Verbesserung in den MC-Klausuren korrelierte in der IG signifikant mit der zeitlichen Beschäftigung mit der App (Spearmans rho=0.741, p=0.004).

**Schlussfolgerung: **Ein App-basiertes Blended-Learning-Konzept führt im Vergleich zum klassischen Lehrkonzept, möglicherweise durch einer intensivere Vor- und Nachbereitung des klinischen Untersuchungskurses, zu einer Verbesserung in den Prüfungsergebnissen.

## 1. Introduction

Proper anamnesis and physical examination of patients represent core competencies of professional medical practice. These skills are the basis for a relationship of trust between doctor and patient and for precise diagnostics and therapy [1]. Up to 70% of suspected diagnoses are made correctly on the basis of taking a patient’s medical history and physical examination [2]. The central role of bedside teaching in medical education as a way to teach these skills is repeatedly emphasized [3], [4], [5]. However, teaching professional behavior and communication with patients [6], medical history taking, clinical examination [7], [8], and basic diagnostic and therapeutic skills to students during practical clinical electives (Famulatur) and during the final practical year of medical study is an enormous challenge.

Studies have shown clear deficiencies in fifth-year medical students in the areas of communicating with patients, taking medical histories, and properly performing physical exams of individual organ systems [9], [10], as well as in the areas of gathering information, giving medical orders, and documenting ward rounds [11]. In one of our own studies it was possible to show that structured changes such as clearly defined learning objectives, the use of course notes for teaching physical examination techniques, and briefing the lecturers led to significant improvement in the student evaluations of “preparation of the teachers”, “structure of the course”, and “self-assessed confidence in performing a physical exam” [12].

New digital learning formats can represent yet another way to improve these core bedside competencies. We have already been able to demonstrate in a study that interactive e-learning cases resulted in improved self-assessments of students regarding history-taking and examination techniques and in diagnostic and therapeutic thinking [13].

To our knowledge, there have been no previous studies on the use of app-based blended-learning approaches in bedside teaching.

The app-based blended-learning approach consists of an app specifically created for the course on anamnesis and examination techniques and an application program for smartphones enabling students to view learning content according to organ systems on their smartphones or mobile tablets and to use the topics to intensively prepare for and review the course content.

The aim of this study was to conduct a randomized controlled pilot study to see if, in the context of a clinical examination course taught at the bedside, an additional app-based blended-learning strategy resulted in (hypothesis a) higher satisfaction and better self-assessments by students when rating their history-taking skills (hypothesis b1) and their ability to perform a physical exam (hypothesis b2), as well as (hypothesis c) better multiple-choice test scores at the end of the course, when compared to a traditional teaching strategy. Also investigated was if the time spent using the app differed from the time spent using the course notes (secondary hypothesis 1) and what influence the time spent using the app or the course notes had on the MC test scores (secondary hypothesis 2).

The results of the pilot study will be initially used to improve the app-based blended-learning approach. In a subsequent main study, after the expansion of the app-based blended-learning tool to include android systems, a skills assessment of the material learned in the clinical examination course will be carried out using an OSCE (Objective Structured Clinical Examination) to better measure students’ communicative and practical skills.

## 2. Methods

### 2.1. Sample

At the Clinic for Heart and Cardiovascular Disease (Klinik für Herz- und Kreislauferkrankungen) at the Deutsches Herzzentrum in Munich and at Medical Clinics I, II, and III of the Klinikum rechts der Isar (university hospitals of the Technical University of Munich), a total of 335 medical students in the first year of the clinical phase of study attended the bedside clinical examination course on a total of six wards during the 2012 summer semester and the 2012/2013 winter semester. This course, which met each week for two hours, covered the following topics: cardiovascular system (**heart**), thorax (**lungs**), **abdomen, lymphatic system** and neoplasms. The students (group size: 3-6 participants) were taught on the different wards by trained instructors who were familiar with both the learning objectives for the clinical examination course and the study.

Due to the type of course app, the pre-requisite for participating in this study was having a specific kind of smartphone or tablet – an “iPhone” or “iPad.” After introducing the study in an email addressed to all students taking the course and encouraging participation, a total of 26 students participated in this study. These 26 students were randomly divided into two groups: an app-based blended-learning intervention group (IG) and a control group (CG) with conventional preparation materials (handouts/course notes). Both groups attended all of the course sessions.

#### 2.2. Study design and procedure

This study involves a prospective, longitudinal, randomized controlled pilot study to compare an app-based blended-learning tool (course app) with a conventional teaching strategy (course notes). Student satisfaction, student self-assessment and written exam-based learning success represent the points of measurement.

**Figure 1:**
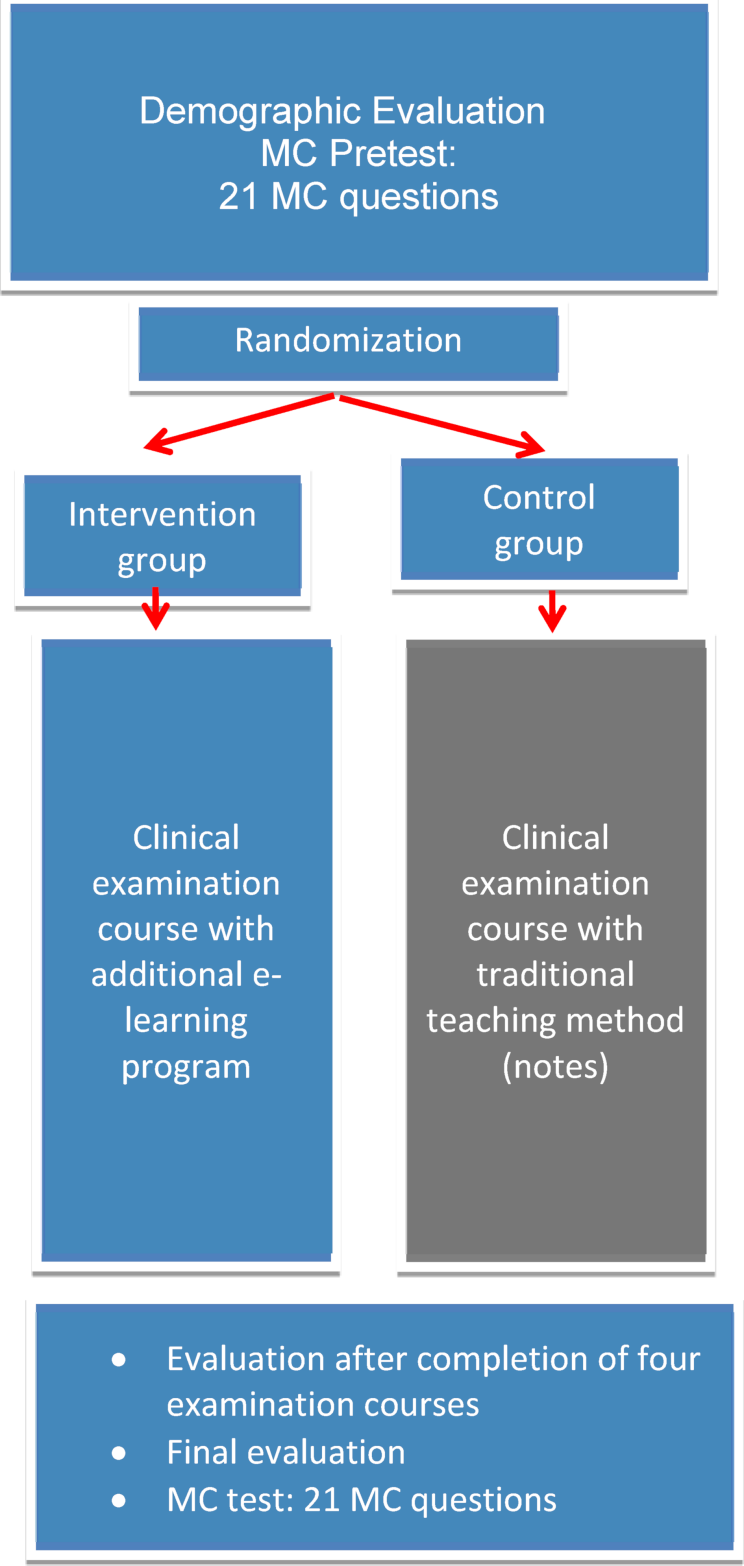
Study procedure

Before the clinical examination course began, a pre-evaluation and a multiple-choice (MC) pretest with 21 MC questions on anamnesis and physical examination were administered online. In the course of the study, all of the students in the IG and CG attended the clinical examination course sessions (1xheart, 1xlungs, 1xabdomen and 1xlymphatic system). At the end of the entire course, the students submitted a final evaluation and took another MC test (21 MC questions) on anamnesis and physical examination. The study procedure is presented in figure 1 (Fig. 1). The study was approved by the Ethics Commission at the Technical University of Munich (Ethics Commission number: 5189-11).

#### 2.3. Blended-learning and conventional teaching strategies

**Figure 2:**
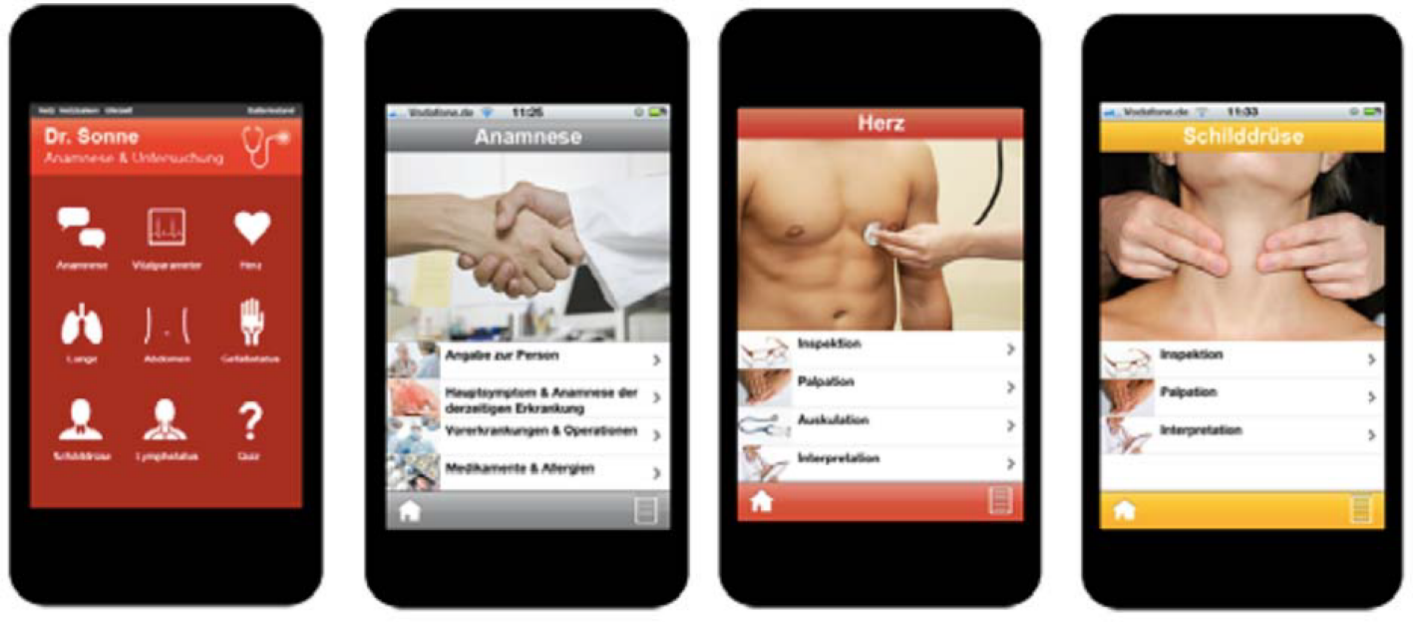
Clinical examination course app (excerpt)

The app-based blended-learning tool consists of an app specifically created for the anamnesis and physical examination course and an application program for smartphones enabling students to view information on their smartphones or tablets and intensively prepare for or review the topics covered in the course. The learning content pertaining to anamnesis and physical examination is presented in the app according to organ systems and explained with descriptive images. The physical examination of each organ system is divided into inspection, auscultation, palpation and interpretation of unusual findings. Use of the app is intuitive with navigation of the content by clicking on the distinctive symbols (see figure 2 (Fig. 2)).

**Figure 3:**
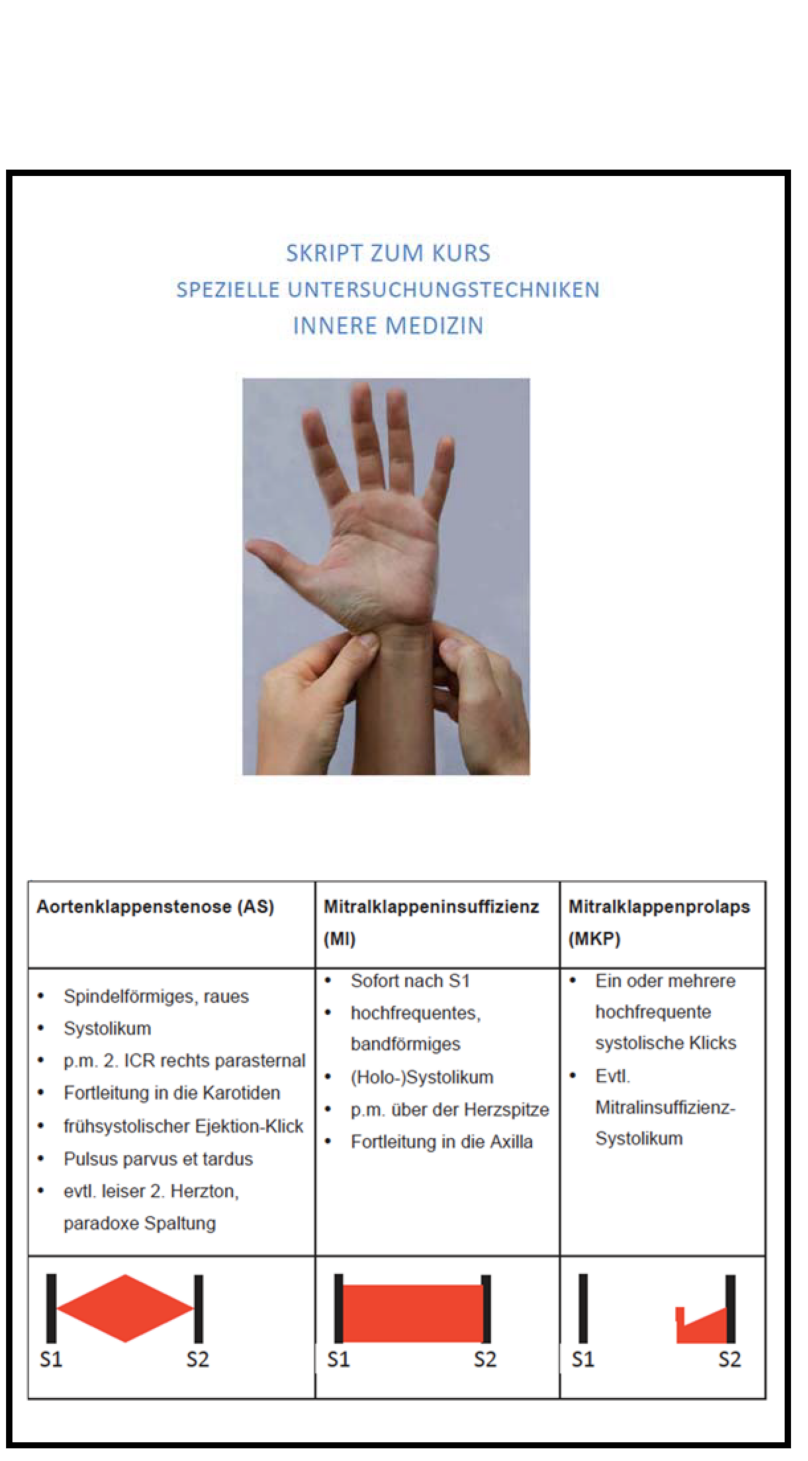
Notes for the clinical examination course (excerpt) – german

In regard to the conventional teaching approach, students received the same information in the form of course notes meant to be used to prepare for and review the material covered in the individual course sessions (see figure 3 (Fig. 3)). In the clinical examination course sessions, the students were taught on each ward by trained instructors who were familiar with both the course learning objectives and the study. The instructors also encouraged the students to regularly prepare for and review courses sessions using the notes or the app depending on which group they belonged to.

#### 2.4. Evaluation

##### 2.4.1. Questionnaire on sociodemographic data

**Figure 4:**
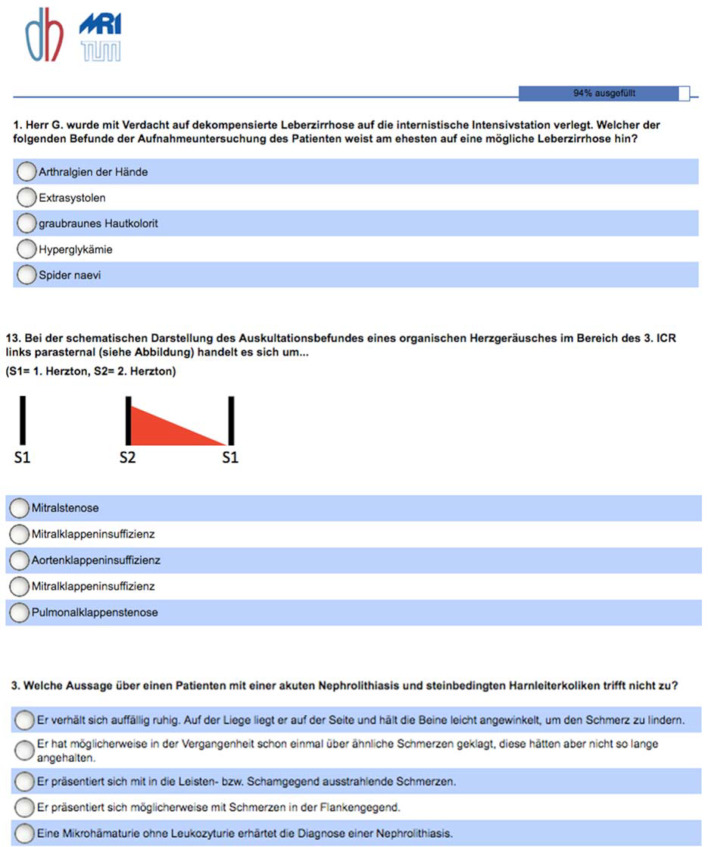
MC test question (excerpt) – german

Information on sociodemographics, grade achieved on the preliminary medical examination (*Physikum*), intensity of digital media use for the purpose of studying, and individual learning strategies was collected on a pre-evaluation using the Sosci-Survey portal [https://www.soscisurvey.de] (see figure 4 (Fig. 4)).

##### 2.4.2. Evaluation of the acceptance of the app and the notes

In the final evaluation, also using Sosci-Survey, students were asked, depending on the group they had been assigned to, if the app or the notes had been helpful in preparing for the course and appealing in their design. Students were also asked if they would like to see more digital media in the future for the purpose of preparing for and reviewing the course’s content. In addition, the students’ self-assessments of their history-taking skills and physical examination skills after completing the course were analyzed. Students rated themselves using the conventional German academic grading scale (grades 1 to 6), whereby 1 is the highest grade and 6 the lowest.

##### 2.4.3. MC test

The MC pretest and MC posttest (each with 21 questions) were administered before and after completing the clinical examination course using online MC tests on the Sosci-Survey portal.

A total of 42 questions on anamnesis and physical examination were randomly divided into pretests and posttests for the online MC tests. All of the MC questions, which had been specifically created based on the information contained in the notes and the app, had only one right answer and were used for the first time in this study.

##### 2.4.4. Longitudinal evaluation of the app and note usage

After receiving weekly email reminders to use the preparation materials, students were also asked how much they had used the app or the notes to prepare for the course (minutes per week, measured as 0, approximately 10, 20, 30, 60, or ≥90 minutes per week).

#### 2.5. Data collection and anonymization

To compile the data and ensure differentiation between the two study arms, the information was at first identifiable using the students’ matriculation numbers. Immediately after the second MC test (at course end) a study employee compiled the data in a table according to matriculation number. After compilation, the matriculation numbers were deleted in all of the sources and replaced by a serial study ID number. The information was fully anonymized as a result.

#### 2.6. Statistical analysis

The sociodemographic data and the data on the evaluation of acceptance were descriptively analyzed and are shown as mean value and standard deviation. The comparison of the demographic variables (in table 1 (Tab. 1)) was done for continuous variables with t tests (two-sided) for independent samples (assuming equal variances) and for dichotomous variables with Fisher’s exact test (two-sided). The comparison of the two groups following the intervention (in table 2 (Tab. 2)) was done with a t test (two-sided) for independent samples.

The statistical analysis of the MC tests was done with the nonparametric Mann-Whitney U test for independent samples to compare the two groups and with a Wilcoxon signed-rank test for related samples to analyze the change in results on the MC posttest in relation to the MC pretest.

The Wilcoxon signed-rank test is a nonparametric test that shows if a difference exists in the central tendency between related samples. In contrast to the t test, the difference of sample means between the samples does not have to be normally distributed. The statistical comparison of the amount of time spent using the app or the course notes was carried out using the nonparametric Mann-Whitney U test for independent samples. The test of secondary hypothesis 2 (influence of amount of time spent using the app or notes on the MC test score) was done using Spearman’s rank correlation coefficient rho (SPSS 17, SPSS Inc. Chicago, IL, USA).

The level of statistical significance was defined as a=5%.

## 3. Results

### 3.1. Sample

Out of the 335 students attending the course on clinical examination techniques at the Clinic for Heart and Cardiovascular Disease at the Deutsches Herzzentrum München and at Medical Clinics I, II and II of the Klinikum rechts der Isar (university hospitals of the Technical University of Munich) in the 2012 summer semester and the 2012/2013 winter semester, a total of 26 students who had a specific type of smartphone or tablet – the “iPhone” or “iPad” – participated in this study (7.8%). The demographic data in table 1 (Tab. 1) shows that no significant differences existed between the groups prior to study begin.

#### 3.2. Response rate

All 26 students (100%) filled out the online pre-evaluation with the questions about demographic characteristics and the online final evaluation with questions about the app, the course notes, and the course itself (see table 1 (Tab. 1) and table 2 (Tab. 2)). The MC pretest and the MC posttest were also taken by all 26 students (100%) (see table 3 (Tab. 3)).

#### 3.3. Acceptance measurement (hypotheses a, b1 and b2)

The results of the acceptance measurement with questions about the app, the course notes, and the clinical examination course in general (hypothesis a), and of the self-assessment by the students regarding their history-taking skills (hypothesis b1) and their performance of physical exams (hypothesis b2) are presented in table 2 (Tab. 2). There were no significant differences between the groups (see table 2 (Tab. 2)).

#### 3.4. MC test scores (hypothesis c)

In both groups there was a significant improvement in the MC test scores after completing the clinical examination course (see table 3 (Tab. 3)).

In contrast to the group that was given course notes, the app group showed a significantly higher improvement in the number of points scored (hypothesis c).

#### 3.5. Longitudinal evaluation of app and note usage (secondary hypothesis 1)

The difference in amount of time (minutes/week) spent using the app in the IG (28.5±33.9) compared to the time spent using the course notes in the CG (7.7±7.3) was not significant (p=0.169).

#### 3.6. Correlation between the IG and CG (secondary hypothesis 2)

**Figure 5:**
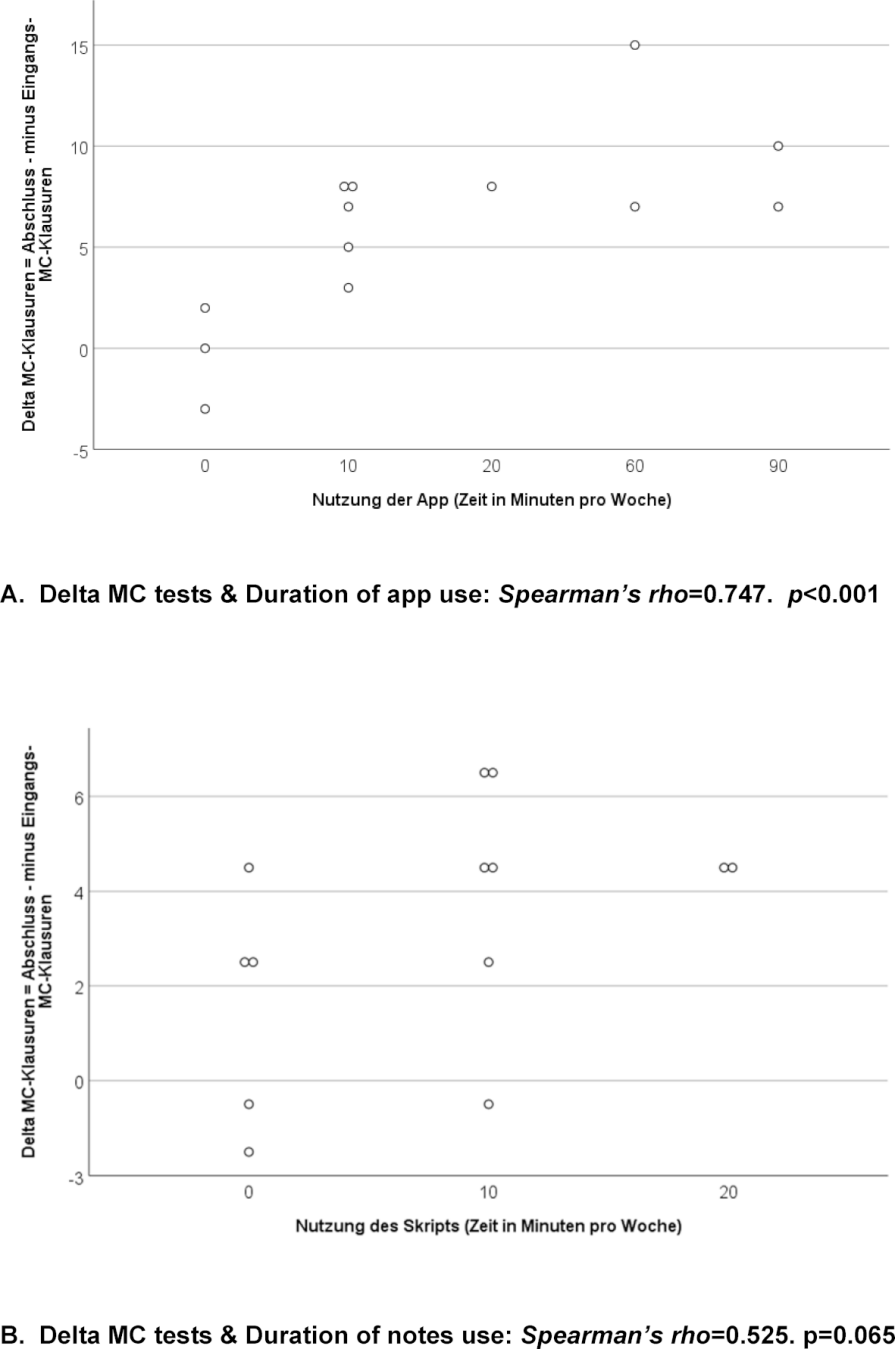
Correlations between use of the app (A) and notes (B) with the change in points scored on the MC tests

The improvement on the MC tests in the IG correlated significantly with the amount of time spent using the app (secondary hypothesis, see figure 5 (Fig. 5)).

## 4. Discussion

The aim of this pilot study was to investigate the value of a structured improvement in teaching by means of a blended-learning strategy in a randomized comparison using a clinical examination course. The “blended-learning tool” consists of an app specifically created for a course on medical history taking and physical examination techniques and an application program for smartphones enabling students to view the course content on their phones and tablets. As part of the conventional teaching method, students were given the same information in the form of course notes to be used to prepare for and review course content. Both strategies resulted in a significant improvement in the scores achieved on the MC posttest in comparison to the MC pretest. However, in comparison to the conventional teaching strategy, the blended-learning strategy led to a significant improvement in the number of points scored on the MC posttest.

Despite the rapid developments in digital teaching and learning in medicine with different successful blended-learning approaches [14], [15], [16], the blended-learning method using an app to prepare for and review the clinical examination course content still remains timely and relevant as a blended-learning approach.

Comparable results to those seen here have been found by several studies looking at the integration of a blended-learning or web-based concept in different areas of medicine [17], [18], [19], [20], [21], [22], [23]. In a meta-analysis, Liu et al. have already been able to show a positive learning effect in the use of blended learning in the health professions [15]; in part, it was even possible – as in the present study – to achieve a greater effectiveness than with nonblended learning methods. Although these claims must be taken conditionally due to the heterogeneity of the studies, it does appear that the concept of blended learning is at least equally effective in comparison with the traditional teaching strategies or with methods that are purely based on e-learning/online learning [15]. Averns et al. have already shown that students who learned musculoskeletal clinical examination techniques using a web-based module performed significantly better on the OSCE than the group of students who prepared using a textbook [17]. The third student group learned the examination techniques in a course setting with demonstration by a tutor. It is interesting to note that the overall result on the OSCE was comparable between the web-based group and the tutor-taught group [17]. Using a combined teaching strategy, as is the case with blended learning, the readiness to engage more deeply with the learning material was encouraged even further [24], [25]. Through the use of up-to-date, appealing and modern media, such the app used here, students were motivated to more thoroughly prepare for and review the course material [24]. This is also reflected in the significant correlation between the amount of time spent using the app and the change in points earned on the MC tests. We believe this could also explain the significantly higher number of points on the final MC test in the blended-learning group. A structured, high-quality e-learning approach in combination with face-to-face teaching appeared to be preferred over the sole use of e-learning or face-to-face teaching [26].

No significant difference was detected between the groups in the self-assessed ability to take a medical history or confidence to perform a physical examination (see evaluation grades). The conventional academic grades “satisfactory” on the self-assessed skills in anamnesis and physical examination after completing the entire clinical examination course, without significant difference between the two groups, showed a lack of confidence regarding very relevant skills that are central to the routine practice of medicine. Fünger et al. have shown in a longitudinal course of 4 semesters a significant improvement in self-assessment regarding skills in performing physical examinations after structured changes to the bedside courses [13]. Despite this, the self-assessment at the end was rated as “good-satisfactory” [13]. In this context, Störmann et al. demonstrated that adequate self-assessment is very difficult for medical students in their final year of study and that almost half rated their abilities to be the opposite of what they were [27]. To acquire sufficient confidence, history taking and properly performing physical examinations require repeated practical training, not only in clinical examination and bedside courses during medical education, but also in practical clinical electives (Famulatur). In addition, the OSCE (Objective Structured Clinical Examination) is more suitable than self-assessment to evaluate practical and communicative skills in history taking and physical examination [27].

Furthermore, no differences were found in the relatively high overall satisfaction of students with the clinical examination course. Thus, it can be stated that, although blended learning contributes in relevant ways to gains in theoretical learning, it cannot replace practical courses [18], [20], [26], [28]. In regard to the app’s technical details, students reported satisfaction with its usability and design. There are various options for applying e-learning strategies to learn practical clinical skills. To date, different web-based methods have been used to learn physical examination techniques [17], [18], [19], [20], [21], [22], [23], [24], [26], [29]. However, we are not aware of any publication that refers to the use of an app. A major advantage to using apps is their availability offline. It is also possible to change and update the learning material at any time. The users can be informed of such changes or updates via push messaging. As a result, the learning content on the app is always up to date and available to the user at all times.

In our study it was also seen that a very high percentage of students in each group in 2012 were still using old exam questions to study. This strategy serves only the long outmoded idea of just learning for tests and does nothing to promote long-term retention of knowledge based on the possible contribution of test-taking to the learning process, as described by Müller et al. [30]. On the questionnaire, all students indicated they wanted to use digital media in the future to prepare for medical courses. This trend can already be seen internationally [15], [20], [23], [26], [28], [29], [31]. Blended learning has also been used in subject areas that are primarily more theoretical, such as pharmacotherapy [25]. Despite the high acceptance and the trend noted above, obstacles to developing, implementing and using such methods are also mentioned. In part, the creation of new e-learning materials has been viewed as very time consuming, above all for medical school faculty members [25]. The inclusion of a computer design expert in the implementation of e-learning materials has been suggested as a possible solution [25], [32]. On the other hand, this can lead to increased costs and the risk that specially developed e-learning tools, in part developed for studies, would not be optimized or used further, especially in German states with little funding [31]. In these cases, the optimization and widespread use of structured and established e-learning strategies would be desirable.

## 5. Limitations

The number of student participants is very low with a total of 26 subjects, and only students who used Apple devices were included in the study. To minimize these influencing factors, students were randomly assigned to one of two study groups. In addition, weekly emails were sent to remind students to record the time spent using the notes or app (minutes per week); however, these emails were retrospective and, as a consequence, were of less importance than the bias of personal memory. Automatic recording of app usage would be better for documenting the time factor. Also, the study measured the gain in theoretical knowledge using only the MC tests, whereas communication and practical skills were not tested. Moreover, it must be pointed out that, due to its ordinal scale, the conventional German academic grading scale is not optimal for self-assessment. Given the multiple comparisons, a Bonferroni correction would have been advantageous, but this was not carried out because of the study’s pilot status. On the basis of the study results, an improved app-based blended-learning tool, expanded to include android devices, will be used in the planned main study and the assessment of skills acquired in the clinical examination course will be done using an OSCE to better measure communication and practical skills. Despite the continued timeliness of app-based blended-learning approaches in medical education, an expansion of the app to include interactive content, videos, and regular updates in the future is viewed as important and is planned.

## 6. Conclusion

Following a blended-learning approach, students achieved better test results in comparison to a conventional teaching method. The blended-learning concept could lead to more in-depth learning of skills among medical students in clinical examination or bedside courses. The students favored the increased integration of digital media in higher education such that other innovative methods should also be used.

## Authors

Shared 1. authorship: C. Sonne and H. Persch.

## Competing interests

The authors declare that they have no competing interests. 

## Figures and Tables

**Table 1:**
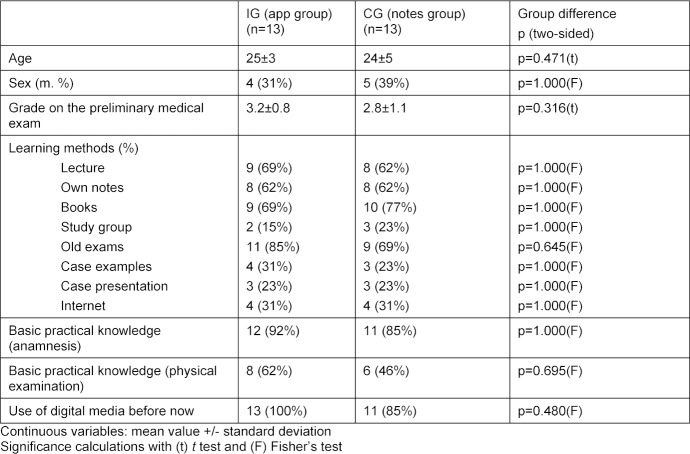
Demographic data for the IG (n=13) and CG (n=13)

**Table 2:**
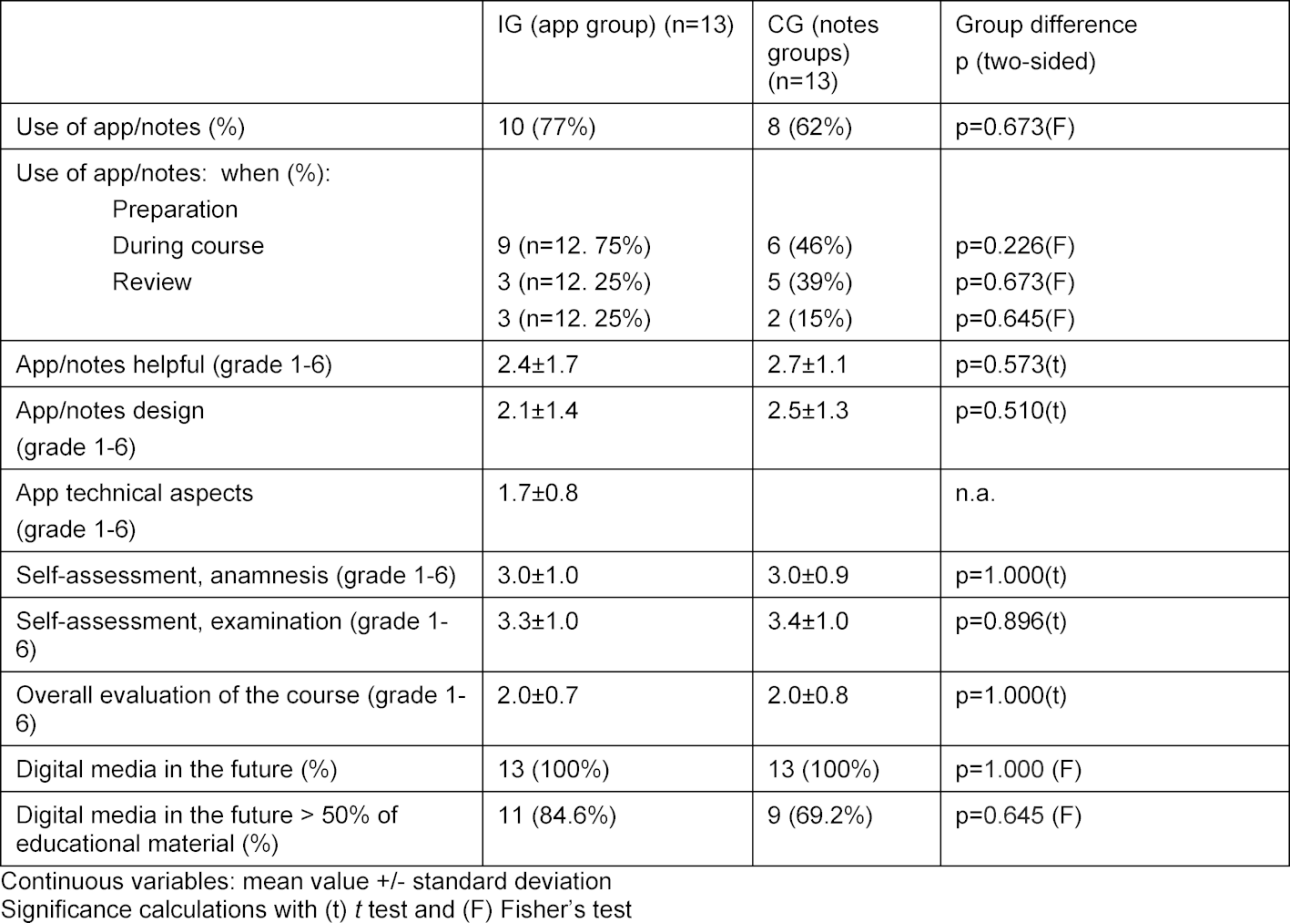
Acceptance measurement in the final evaluation IG (n=13) and CG (n=13) after completing the clinical examination course (n and % and evaluation using the German academic grading scale, 1 through 6)

**Table 3:**
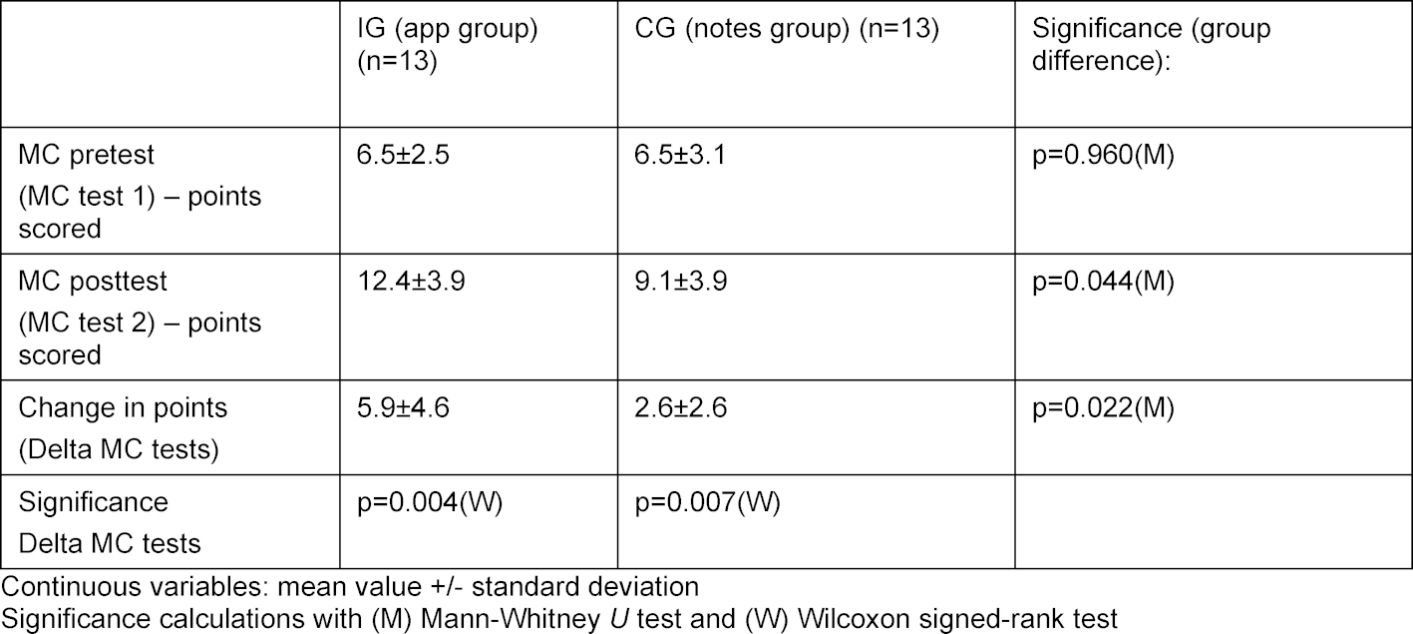
Results on the MC tests in the IG (n=13) and the CG (n=13) before and after completion of the clinical examination course (points correspond to correct answers to 21 questions on the pretest and 21 questions on the posttest)
